# Enzyme replacement therapy for lysosomal storage disorders in India

**DOI:** 10.1186/1755-8166-7-S1-I29

**Published:** 2014-01-21

**Authors:** Mamta Muranjan

**Affiliations:** 1 Genetic Division, Depart of Pediatrics, KEM Hospital, Parel, Mumbai-400012, India

## 

Lysosomal storage disorders (LSD) are a heterogeneous group of inherited metabolic disorders with a combined frequency of 1 in 5000 affected live births. The commonest pathogenic mechanism is qualitative or quantitative deficiency of one of the 50 known lysosomal enzymes (acid hydrolases) involved in the catabolism of a variety of molecules. The outcome of this deficiency is progressive accumulation of partially degraded compounds which stealthily leads to multiorgan dysfunction.

Unlike many inherited metabolic disorders, diet plays no role in the control of LSD. However treatment in the form of Enzyme replacement therapy (ERT) is available for a few disorders. The diseases for which ERT is currently the standard of care are Gaucher disease (Imiglucerase, Velaglucerase, Taliglucerase), MPS I (Laronidase), MPS II (Idursulfase), Pompe disease (Alglucosidase alpha), Fabry disease (Agalsidase beta, Agalsidase alfa) and MPS VI (Galsulfase). These drugs are human recombinant products manufactured in in-vitro tissue culture systems. ERT alters the natural history of these diseases and reverses many of the symptoms. However, as the drugs do not penetrate the blood-brain barrier, there is no impact on CNS disease.

Gaucher disease was the first LSD to be treated with ERT in India since 1999-2000. Six centres in India located in Mumbai, Delhi, Lucknow and Chennai are treating patients with ERT.

The major factor limiting widespread access to ERT in the country is the prohibitive cost, the need to import the drugs and lack of infrastructure for comprehensive evaluation and supportive care. Access has been facilitated for Indian patients through a charitable access program (INCAP). A board of seven National experts and a panel of International experts constitute the Indian Medical Advisory Board (IMAB) to screen patients for eligibility on the basis of pre-determined objective criteria. The experts also provide guidance for evaluation and management of LSD. To date approximately 60 individuals with Gaucher diseases are receiving ERT in India. Some of these have been receiving ERT for more than 10 years and are young adults pursuing graduate level academics. Additionally, 20 patients with MPS I, two with Fabry and 20 with classic infantile Pompe are receiving ERT. ERT is currently not available for MPS II and MPS VI in India.

Twelve individuals with Gaucher disease (10 with type I) have been treated with Imiglucerase. Of these, one with severe disease resulting in hepato-pulmonary syndrome expired and one was transitioned to oral substrate reduction therapy.

